# Dietary Lipid and Cholesterol Induce Ovarian Dysfunction and Abnormal LH Response to Stimulation in Rabbits

**DOI:** 10.1371/journal.pone.0063101

**Published:** 2013-05-14

**Authors:** Anne-Gaël Cordier, Pauline Léveillé, Charlotte Dupont, Anne Tarrade, Olivier Picone, Thibaut Larcher, Michèle Dahirel, Elodie Poumerol, Béatrice Mandon-Pepin, Rachel Lévy, Pascale Chavatte-Palmer

**Affiliations:** 1 INRA, UMR1198 Biologie du Développement et Reproduction, Jouy-en-Josas, France; 2 APHP, Hosp Antoine Béclère, Service de Gynécologie-Obstétrique et Médecine de la Reproduction, Clamart, France; 3 Université Paris 13, Sorbonne Paris Cité, Unité de Recherche en Epidémiologie Nutritionnelle (UREN), Bobigny, France; 4 APHP, Hôpital Jean-Verdier, Bondy, France; 5 PremUp foundation, Paris, France; 6 INRA, UMR 703 APEX, Oniris, Nantes, France; Imperial College London, United Kingdom

## Abstract

**Background/Aim:**

Excess of fat intake is dramatically increasing in women of childbearing age and results in numerous health complications, including reproductive disorders. Using rabbit does as a biomedical model, the aim of this study was to evaluate onset of puberty, endocrine responses to stimulation and ovarian follicular maturation in females fed a high fat high cholesterol diet (HH diet) from 10 weeks of age (i.e., 2 weeks before normal onset of puberty) or a control diet (C diet).

**Methodology/Principal Findings:**

Three experiments were performed, each including 8 treated (HH group) and 8 control (C group) does. In experiment 1, the endocrine response to Gonadotropin releasing hormone (GnRH) was evaluated at 13, 18 and 22 weeks of age. In experiment 2, the follicular population was counted in ovaries of adult females (18 weeks of age). In experiment 3, the LH response to mating and steroid profiles throughout gestation were evaluated at 18 weeks of age. Fetal growth was monitored by ultrasound and offspring birth weight was recorded. Data showed a significantly higher Luteinizing hormone (LH) response after induction of ovulation at 13 weeks of age in the HH group. There was no difference at 18 weeks, but at 22 weeks, the LH response to GnRH was significantly reduced in the HH group. The number of atretic follicles was significantly increased and the number of antral follicles significantly reduced in HH does vs. controls. During gestation, the HH diet induced intra-uterine growth retardation (IUGR).

**Conclusion:**

The HH diet administered from before puberty onwards affected onset of puberty, follicular growth, hormonal responses to breeding and GnRH stimulation in relation to age and lead to fetal IUGR.

## Introduction

Adult lifestyle - generally diet and sedentary habits – as well as environmental chemicals are known factors impacting the fertility of men and women. According to the recent national observational study “Obésité-Epidémiologie” (ObEpi), the prevalence of overweight (defined by a Body Mass Index (BMI) >25 kg/m^2^) and obesity (BMI >30 kg/m^2^) among French women is 25% and 15% respectively [1]. The weight of women in childbearing age is dramatically increasing by about 0.5 to 0.7 kg/year. Moreover, in Europe, lipid intake represents more than 32% of food intake with a high proportion of saturated fat [2]. The Normal Weight Obese syndrome, which affects up to 37% of apparently healthy patients, is characterized by a BMI <25 together with a high fat mass (>30%), leading to high inflammatory cytokines and a high level of oxidative stress [3], [4]. Oxidative stress is well known to be detrimental in many tissues, including the female reproductive tract [5]. Compromised oocyte quality, altered pubertal development and hormonal and ovulatory dysfunction have been described.

Early onset of puberty has also been described in animals fed a high fat diet or in models of obese animals. Feng Li et al. [6] showed a dramatic acceleration of the LH pulse frequency concomitant with an early onset of puberty in rat fed a high fat diet. Even the short term (7 days) administration of a fat enriched diet can significantly modify endocrine responses, inducing higher plasma LH concentrations in rats [7]. In pigs, Iberian gilts, which are naturally obese due to leptin resistance subsequent to a mutation in the leptin receptor, have an earlier onset of puberty compared to other breeds [8]. In primates, precocious menarche was reported in rhesus monkeys fed a high calorie diet, in association with high nocturnal plasma LH concentrations [9].

In adults, numerous studies have demonstrated a reduced LH surge in adult obese women. Overweight and obese women were found to have a longer follicular phase and reduced plasma LH concentrations [10], [11], [12]. In a study where 22 fertile, overweight or obese women were compared with 10 fertile, normal-weight women, the overweight group had lower plasma LH concentrations compared with normal weight women and LH concentrations were negatively correlated to BMI and waist circumference [13]. An additional study including 18 premenopausal, eumenorrheic (nonpolycystic syndrome) morbidly obese women and 12 eumenorrheic, normal-weight subjects found a dramatic reduction of both the amplitude and the mean LH concentration in the morbid obesity group [14]. In women, it has been suggested that the pituitary response to endogenous GnRH is attenuated by obesity. Recent work in Assisted Reproductive Technologies (ART) has shown that obese patient require higher doses of gonadotropins and prolonged ovarian stimulation compared to normal weight individuals [15], [16], [17]. Other studies, however, have failed to demonstrate a difference in the ovarian response to stimulation in obese women [18], [19], [20].

Effects of high fat diets were also observed on the ovarian function. In rats, the administration of a cafeteria diet was shown to negatively affect female reproduction by reducing the number of oocytes (median number (interquartile range) of oocytes in cafeteria diet fed females [1(0/6)] vs. chow-fed rats [10(8/12)]) and preantral follicles in the ovary [21]. In obese hyperinsulinemic (fa/fa) adult rats, the ovaries from the obese rat contained more corpora lutea, antral, pre antral and atretic follicles compared to controls, with a positive association between follicular atresia and the expression of the pro apopotic factor of transcription FOXO1 [22]. Similarly, the number of follicles present in the ovary of obese, ob/ob mice, is reduced and granulosa cell apoptosis and follicular atresia are increased [23].

In the same model, excessive lipid storage was shown to induce ovarian function disorders with advanced follicular atresia, apoptosis and defective steroidogenesis [24]. In contrast to what has been found in rodents, fat supplementation in lactating cows did not appear to affect follicular growth, although luteal progesterone was reduced in supplemented cows [25]. In the obese Iberian pig model, the ovarian follicular population, plasma estradiol concentrations and ovulation rates were not different compared to lean pigs [26].

High fat diets appear to induce different effects on reproduction according to the model, the age, the exposure and/or the fat content in the diet. The objective of this work was to explore the effects of a high fat, high cholesterol diet administered from the prepubertal period on the onset of reproductive function, endocrine status and follicular growth, using a previously established rabbit model [27]. The rabbit was chosen as a model because of its decisive advantages over the rat or mouse models for the proposed longitudinal studies [28]. Indeed, it is a preferred model for diet-induced lipid metabolic disorders and insulin resistance [29] and its size allows for repetitive blood sampling and transabdominal ultrasound during pregnancy.

## Materials and Methods

### Ethical Statement

The experiment was performed in accordance with the International Guiding Principles for Biomedical Research involving Animals as promulgated by the Society for the Study of Reproduction and in accordance with the European Convention on Animal experimentation. The animal studies were approved by the local animal care and use committee (CSU UCEA) and received ethical approval from the local ethics committee (COMETHEA), under protocol number 12/029. Researchers involved in the work with the animals possessed an animal experimentation license (level 1 or 2) delivered by the French veterinary authorities.

### Animals and Diet

Forty-eight female New Zealand rabbits (INRA 1077 or PS 19 line) were housed individually with free access to water, under a 8 hours light/16 hours dark photoperiod unless stated below. At 10 weeks of age, they were allocated to one of two groups and fed *ad libitum* with either a hyperlipidic hypercholesterolemic diet (HH group) (n = 24) or a control diet (C group) (n = 24) containing respectively 7.71% or 1.83% fat and 0.2% or 0% cholesterol as previously described [27].

The fat supplementation consisted of soybean oil, i.e. mainly polyunsaturated fatty acids (N6/N3 = 6.86). The euthanasia of the animals was performed by exsanguination after electronarcosis at the local experimental slaughterhouse, according to the protocol approved by the local ethics committee and the veterinary services.

### Experimental Protocol

**Figure 1:**
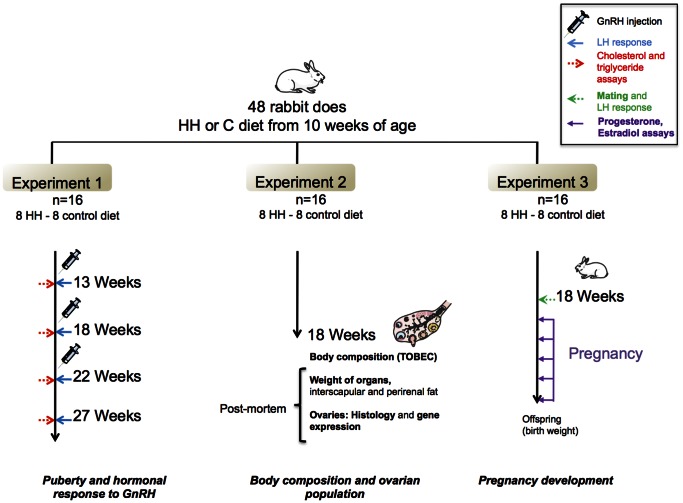
Schematic representation of the experimental protocol. Three experiments were performed, each including 8 treated (HH group) and 8 controls does (C group). Experiment 1: Influence of HH diet on puberty and hormonal response. Experiment 2: Influence of HH diet on the ovarian follicular population. Experiment 3: Influence of HH diet on endocrine function during gestation, with fetal growth monitored by ultrasound and recording of offspring birth weight.

The study was organized in 3 consecutive experiments as summarized in Figure 1.

#### Experiment 1 (n = 16)

The first experiment was conducted in does from 10 to 27 weeks of age to evaluate the influence of the HH diet on the onset of puberty and ovulation disorders according to age. Experiments were repeated in the same animals at 13, 18 and 22 weeks of age.

Females were fasted overnight, weighed and blood was collected from the auricular vein into EDTA coated vacutainers for biochemical dosages (total cholesterol and triglycerides, progesterone, leptin and estradiol).

The rabbit being an induced ovulator, puberty can only be assessed by inducing ovulation, either by mating or by chemical stimulation. Here, the onset of puberty was evaluated by injecting 40 µg of a GnRH analogue (Receptal®) IM after 1 week of synchronization with light (16 hours of light/8 hours of dark). Blood was collected for LH assay at 0, 30, 60, 90, 120 and 180 minutes after injection. Blood samples were obtained through a catheter previously placed in the peripheral ear vein into EDTA coated vacutainers which were placed on ice until processing. Samples were centrifuged within an hour of collection and supernatants stored in several aliquots at −20°C until analysis.

#### Experiment 2 (n = 16)

In the second experiment, ovaries were collected from 18 week old females after slaughter in order to assess follicular populations in the ovaries and ovarian function.

One week before slaughter, 16 does were synchronised by 16 hours of light and 8 hours of dark by day. At 18 weeks of age, the body composition was analysed by Total Body Electrical Conductivity (TOBEC) [30]. After euthanasia, the liver, kidney, ovaries, interscapular and perirenal fat were weighted. All ovaries were flash frozen in liquid nitrogen and then conserved at −80°C for molecular analysis (1 ovary per animal) or fixed in 10% formalin and processed for histological analysis (1 ovary per animal).

For histological analysis, ovaries were embedded in paraffin wax. Four transversal 6 µm sections, each spaced from 100 µm minimum from the others, were stained per ovary using a routine hematoxylin-eosin-saffron (HES). The microscopic observation was performed blindly in two steps: 16 randomly selected fields per ovary were observed with ×10 magnification (4 fields per section, 4 sections per ovary) and 4 randomly selected fields at x5 magnification per ovary (2 fields per section, 2 sections per ovary) using a light microscope combined with a digital camera (DXM 1200, Nikon, Champigny, France). The intermediate magnification (×10) was chosen in order to count all smaller follicles (primordial, primary, secondary) and low magnification (×5) to count larger ones (tertiary, hemorrhagic, luteum corpus). Bodensteiner histological criteria were used for follicle classification [31]. Atretic follicles as assessed by the irregular shape of the pellucid membrane, were also numbered. Repeatability was tested by reproducing measurements 3 times on the same sample by the same experimentator.

The expression of 9 genes involved in ovarian development was studied by RT-qPCR. Total rabbit RNAs were extracted from each sample using Trizol® reagent (Invitrogen Life Technologies, Cergy-Pontoise, France) using the RNeasy Mini kit (QIAGEN SA, Courtaboeuf, France), following the manufacturer’s instructions. RT was performed on each sample using 5 µg Dnase-treated RNA incubated with random hexanucleotide primers with Superscript II (Invitrogen, Cergy-Pontoise, France), according to the manufacturer’s instructions.

Real-time PCR analysis of the different genes was performed using the ABIPrism 7700 HT apparatus (Applied Biosystems). Briefly, PCR was performed in triplicate with the ABsolute blue QPCR SYBR Green ROX mix (Abgene, Les Ulis,France), using 50 ng of cDNA from the RT. Specific primers were used (data supplied as Data S1). Control experiments were performed to ensure that the primers could not amplify any genomic products. Cycle conditions were as follows: one cycle at 50°C for 2 min, followed by 1 cycle at 95°C for 10 min, followed by 45 cycles at 95°c for 15 s and 60°C for 1 min. All expression data were normalized using the mean expression level for each sample of three different genes (H2AFX, CPR2 and YWHAZ). Results were analyzed using Qbase Software (Ghent University, Ghent, Belgium). Each condition (control or HH diet) represents the mean of 5 different animals.

#### Experiment 3 (n = 16)

The aim of the third experiment was to measure the effects of the HH diet on hormonal response just after mating and during gestation. At 18 weeks of age and after synchronization with light as described in Experiment 2, 8 control and 8 HH rabbit does were mated with 3 different males. The LH response was analysed in samples collected from the peripheral ear vein, through a previously placed catheter, into EDTA coated vacutainers, 0, 30, 60, 90, 120, 180, 240, 300, 360 minutes after mating. Blood samples were collected from the peripheral ear vein for estradiol (E2) and progesterone (P4) assays 3, 6, 13, 20 and 27 days after mating.

Pregnancy was followed by ultrasound scanning at 13, 21 and 27 days of gestation as described previously [27], [32], using a Voluson V8 (General Electrics Healthcare). Does were allowed to deliver naturally and offspring were numbered and weighed at birth.

Total cholesterol and triglycerides were analysed using a colorimetric enzymatic technique (OSR 6116-6187-61118 (OLYMPUS, Hamburg, Germany)).

Hormones were assayed in one single assay to avoid inter-assay variability. LH was measured both after stimulation (experiment 1) or mating (experiment 3) with a multispecies ELISA, with an intra-assay variation of 2.5% for values above 1 ng/ml (LH-DETECT®, Repro-Pharm, France). Leptin was measured in duplicate with a multi-species leptin RIA Kit (LINCO Research, Missouri, USA) (25 µL) with an intra-assay variation below 5% [33]. Estradiol was measured in duplicate by using the I^125^ E2 Diasorin RIA Kit with an intra-assay of 8% (Sorin diagnostic, Antony, France). Progesterone was measured in duplicate (25 and 40 µL) by direct RIA method (without extraction) using an in-house antibody. Intra-assay variation was 25%.

### Statistical Analyses

Non parametric statistical analyses were used. For LH response, areas under the curves (AUCs) were calculated for each animal and data analysed using the Mann and Whitney test. The others variables were analysed by a comparison of means using the Wilcoxon test (PROC NPAR1WAY, SAS version 9.1; SAS Institute, Cary, NC). For progesterone and estradiol values, rabbits were also classified according to the peak value time and a Fisher’s test was used to analyse repartition. All results are expressed as means ± Standard Error of the Mean (SEM) (figures as curves) or as median (quartile1; quartile3) (box plot figures). Significance was defined as *P≤*0.05.

## Results

### Experiment 1

#### Weight and metabolic blood test results

The HH diet did not induce obesity, as no significant difference in body weight was observed at 13, 18, 22 and 27 weeks (data supplied as Data S2).

Plasma cholesterol concentrations were significantly higher in HH does compared to controls, at all times. However, no significant difference was found between HH and Controls for plasma triglyceride concentrations (data supplied as Data S3 and S4).

#### Hormonal response after induction of ovulation

**Figure 2:**
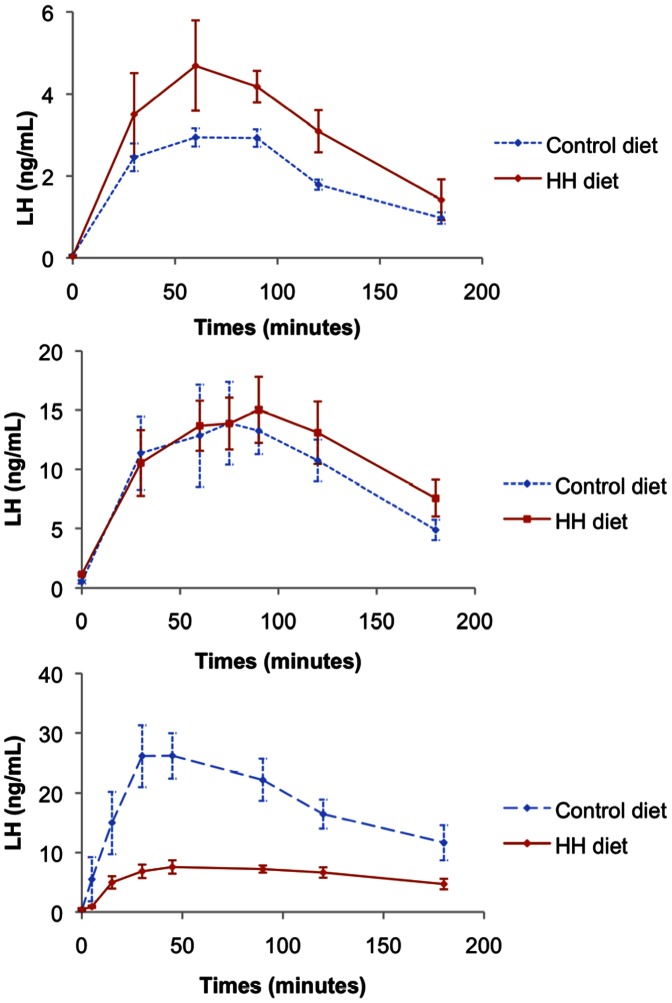
Mean (±SEM) LH response after induction of ovulation at 13 (A), 18 (B) and 22 weeks (C) of age (ng/ml).

The LH response to GnRH stimulation according to age is shown in Figure 2. At 13 weeks, the peak LH concentrations in response to GnRH was significantly higher in HH does (*P*<0.02). At 18 weeks, no difference was found between the two groups (*P* = 0.428). At 22 weeks, the LH response was significantly reduced in HH does (P<0.0001).

**Figure 3:**
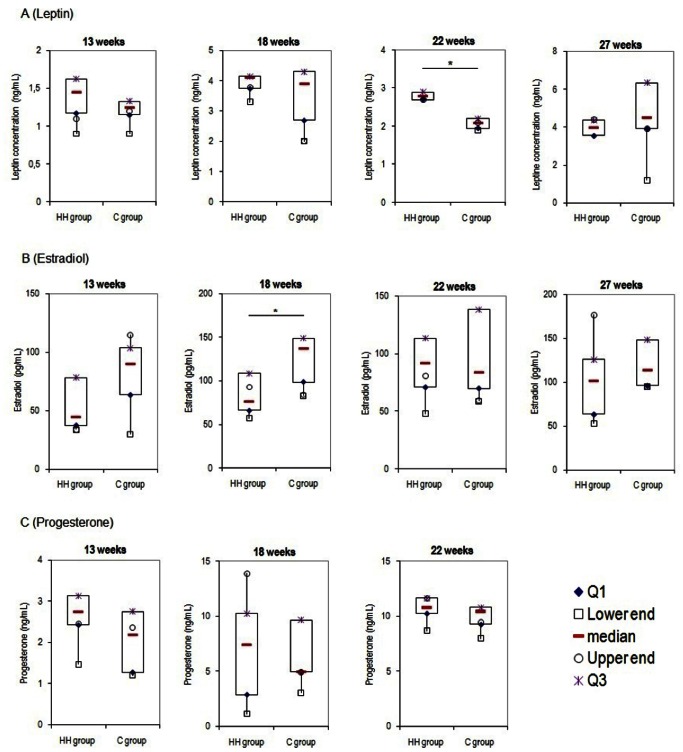
Mean (±SEM) serum leptin, estradiol and progesterone concentrations according to age in HH and Control does. Each box plot represents the distribution of values in each group at 13, 18, 22 and 27 weeks of age for leptin and estradiol (A and B) and at 13, 18 and 22 weeks of age for progesterone (C). Median values are indicated by the red line within the box. The upper point (in purple) and the lower point (in blue) represent the first and the third quartile respectively. The highest and the lowest values are representing by circle and square respectively. *indicates *P*<0.05.

Leptin serum concentrations were significantly higher in HH does compared to controls at 22 weeks of age (2.8 ng/ml (2.7;2.9) vs. 2.1 ng/ml (2.0;2.2), respectively) *P*<0.05) but not before (Figure 3 (A)). Estradiol concentrations were significantly reduced in HH does at 18 weeks of age (76.0 pg/mL (66.0;108.5) vs. 137.0 pg/mL (98.5;149.0) respectively, *P*<0.05) (Figure 3 (B)). No difference was found between groups for progesterone concentrations at any time (Figure 3 (C)).

### Experiment 2

#### Body composition by TOBEC

At 18 weeks of age, HH does had a significantly higher body lipid content compared with controls (6.11±0.19% vs. 5.67±0.14%, respectively, *P* = 0.04, other data supplied as Data S5).

Despite a large difference in the means for adipose tissue weight (mean perirenal+interscapular tissue: 101.84 g ±15.41 vs. 82.95 g ±16.90 in HH and Controls, respectively, *P* = 0.20), there was no significant difference in adipose tissue weight. There was no significant difference in organ weight either (liver, kidney and ovaries), data supplied as Data S5).

#### Histological results

**Figure 4:**
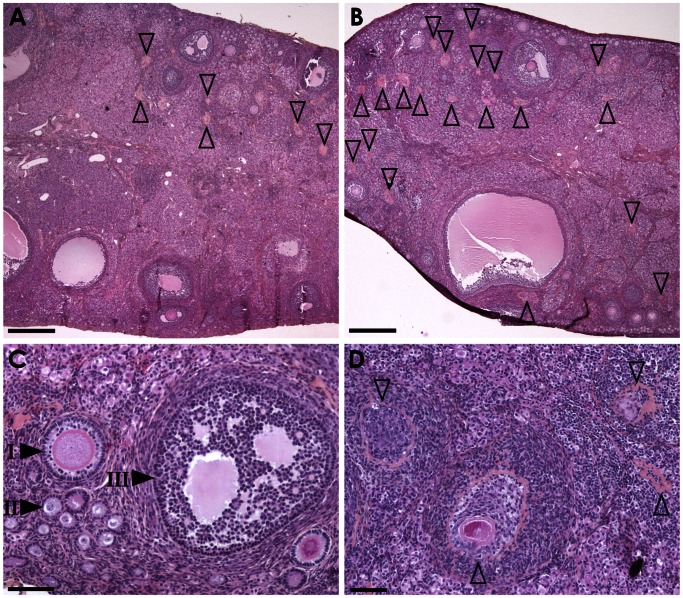
Ovarian histology. Hematoxylin-eosin-saffron staining ovary sections of rabbits fed a control diet (left panel) or HH diet (right panel) at low (A,B) or high (C,D) magnification. Compared to the control sample (A), numerous atretic follicle remnants (open arrowheads) are scattered in the ovary parenchyma of the high fat diet-fed animal (B). With higher magnification, primary (I), secondary (II) and tertiary follicles (III) are observed in the control samples (C) whereas numerous fields in high fat diet-fed animal samples are devoid of maturing follicles and are only composed of atretic follicle remnants at different stage of involution (D). Scale bars = 500 µm (A, B) and 100 µm (C, D).

The aim of this analysis was to evaluate follicular populations in the ovary. Using intermediate ×10 magnification, a significantly higher number of atretic follicles was observed in the HH group compared to controls (51.40±4.83 vs. 39.10±5.02, respectively, *P* = 0.05) (Table 1, Figure 4). Using the x5 magnification, a significantly reduced number of antral follicles was observed in the HH group compared to controls (7.38±1.00 vs. 17.88±2.28, respectively, P<0.001) (Table 2, Figure 4).

**Table 1:** Mean number follicles (intermediate magnification).

Type of follicle	HH diet	Control diet	*P*
Primordial	289.00±29.64	340.20±37.05	0.14
Type I	30.60±3.51	29.70±3.91	0.43
Small type II	16.70±2.48	14.70±3.24	0.31
Large type II	12.00±2.42	13.50±2.18	0.32
Antral	9.50±1.85	12.20±1.49	0.13
Atretic	51.40±4.83	39.10±5.02	0.05
Hemorrhagic	3.60±0.65	6.20±0.96	0.02
Corpus luteum	0.00±0.00	0.12±0.13	0.16
Corpus albicans	0.60±0.26	0.25±0.16	0.12

Follicles are classified in 9 categories. The counting was realized using one ovary per rabbit and per group (intermediate magnification).

**Table 2:** Mean number follicles according to size (low magnification).

Type of follicle	HH diet	Control diet	*P*
Type II	15.50±2.27	12.13±2.01	0.14
Antral	7.38±1.00	17.88±2.28	<0.001
Atretic	44.38±8.27	31.13±4.89	0.09
Hemorrhagic	3.13±0.61	2.13±0.69	0.14
Corpus luteum	0.00±0.00	0.25±0.16	0.07

Follicles are classified into 5 categories. The counting was realized using one ovary per rabbit and per group (low magnification).

#### Gene expression in the ovary

The expression of 9 genes involved in ovarian development was studied by RT-qPCR. These transcripts fell within functional categories which included: (i) steroidogenesis (HSD3B2), (ii) germ cell differentiation (VASA), (iii) apoptosis (Caspase), (iv) folliculogenesis (FOXL2, FST, GDF9, BMP15), and (v) receptors (ESR1, ESR2). Of these quantified transcripts, none showed any significant difference in expression between the 2 groups (data supplied as Data S6).

### Experiment 3

#### Endocrine response after mating

**Figure 5:**
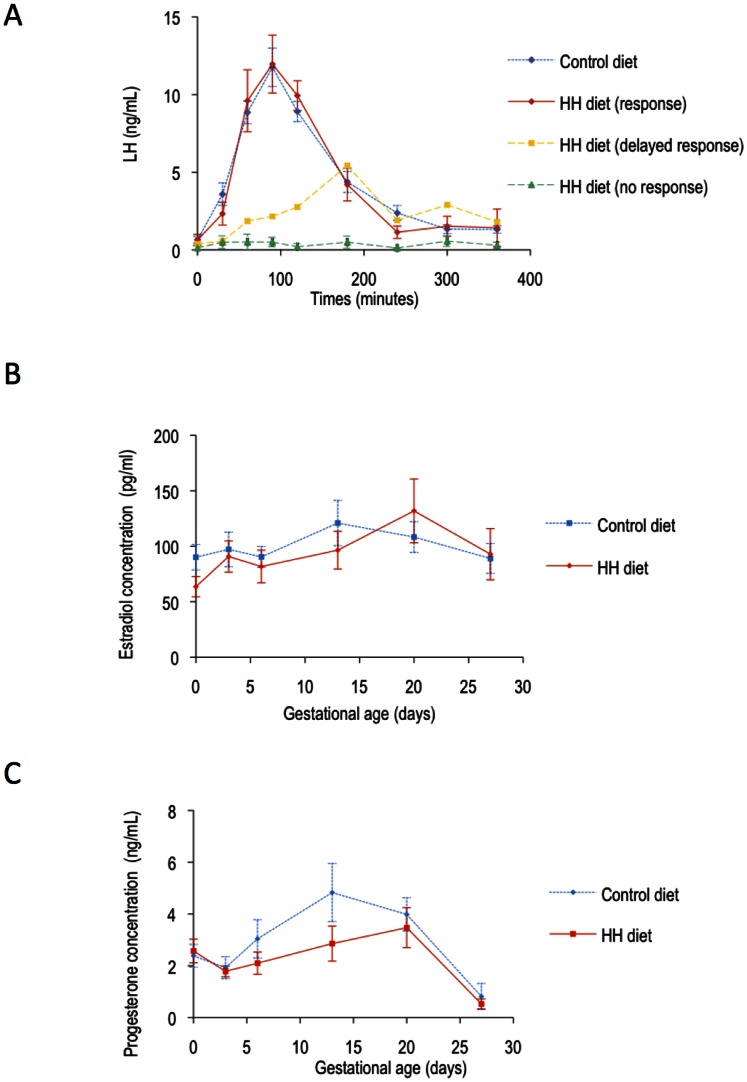
Hormonal response (LH response, estradiol and progesterone) after mating. (A) Mean ±SEM serum LH concentrations (ng/mL) according to time after mating and response to mating: C animals that all responded (N = 7). HH animals that had a LH response (N = 5). – HH animal that had a delayed response (N = 1). – HH animals that did not respond (N = 2). (B) Mean ±SEM serum estradiol concentrations (pg/mL) in HH and C does at 0, 3, 6, 13, 20 and 27 days of gestation (term: 31 days). (C) Mean ±SEM serum progesterone concentration (ng/mL) in HH and C does at 0, 3, 6, 13, 20 and 27 days of gestation (term: 31 days).

After mating at 18 weeks of age, all the control does but only 4 out of 7 HH does had a normal LH response. The three other animals had either no LH increase at all (N = 2) or a delayed response (N = 1) (Figure 5 (A)) but the difference was not significant between the two groups.

During gestation, there was no significant difference between the groups for plasma estradiol and progesterone concentrations, although the hormonal peaks appeared to be delayed in HH does (Figure 5 (B and C)).

#### Fetal growth and offspring characteristics

All control females became pregnant whereas the two females that had no LH response did not become pregnant. The median litter size was 6 pups in the Control group and 7 pups in the HH group, with no statistical difference between groups.

On ultrasound examination, biparietal diameter, abdominal circumference and body surface area were significantly smaller in HH diet fetuses at 27 days of gestation (*P* = 0.012, *P* = 0.001; *P* = 0.005 respectively) (data supplied as Data S7). At birth, HH pups were significantly lighter in the HH group compared to control group (37.3±1.89 g vs. 47.77±1.63 g, respectively, *P*<0.0001).

## Discussion

This study evaluated the effect of a diet supplemented in soybean oil and cholesterol, administered from before the age of puberty (13 weeks) on reproductive hormones, ovarian maturation and ovulation disorders, using a previously used rabbit model. In summary, although the rabbit does were not overweight, the HH diet administered from before puberty onwards affected onset of puberty, follicular growth and hormonal responses to breeding and GnRH stimulation. Although the number of antral follicles was decreased and that of atretic follicles increased in HH does, the ovarian expression of genes involved in folliculogenesis was not modified. Fertility and prolificity did not appear to be affected, although 2 does did not get pregnant as a result of a lack of LH response to mating (LH response and ovulation are induced by mating in rabbits). In contrast, fetal growth was affected with intra-uterine growth retardation observed in offspring, as previously observed [27].

The enhanced LH response at 13 weeks in the HH group indicates an early puberty onset compared to controls. An early onset of puberty [9] and of high LH pulse frequency [34], [35] has also been demonstrated in obese rats. In this model, a fat-related signal has been shown to facilitate the activation of hypothalamic GnRH release and advance the onset of puberty [6], [36]. In rhesus monkeys, administration of a high-calorie diet results in the acceleration of growth accompanied with precocious menarche [6]. In humans, the onset of puberty is influenced mainly by genetics, lifestyle, environment, nutrition and body fat [37], [38], [39]. In adults, body size parameters, such as weight or BMI, are strongly correlated with an earlier onset of puberty [37]. In the prepubertal age (5–9 years), increased subcutaneous fat and BMI are associated with increased likelihood of early (<11 years) menarche [38]. Interestingly, the studies evaluating whether nutritional habits (total, unsaturated or saturated fatty acids) could influence age of menarche are still controversial. Some studies reported that higher intake of total fat or PUFA intake in childhood were associated with earlier age at menarche [39], [40], [41], [42] while others found that a balance towards satured fat or MUFA decreased the risk of early menarche [41], [43]. In any case, early menarche constitutes a robust marker of obesity and mortality risk in adult life [44], [45]. In humans, an earlier age of menarche has been associated with an earlier age at menopause [46], [47]. In experiment 3, at 18 weeks of age, although there was no significant difference in the LH response to GnRH, 2 out of 8 HH does did not respond to mating and 1/8 had a delayed response. In experiment 1, in slightly older animals, at 22 weeks of age, the HH diet induced a significant decrease of the LH response in all females after induction of ovulation. In rats, a reduced LH surge before estrous and a reduction in plasma estradiol leading to anovulation were reported in females fed a high fat diet (45% calories from fat) [48]. In humans, a significant reduction of both amplitude and/or mean LH has been reported in obese compared to normal weight women [10], [11], [12], [14] and a recent study on 154 normal weight and 25 obese weight women showed that adiposity may delay the timing and the concentration of hormonal peaks (progesterone and LH) during the menstrual cycle [13]. In mice, neonatally undernourished is associated with delayed puberty and an impairment of the peripubertal GnRH/LH system to respond to ovariectomy [49].

Moreover, in the present study, circulating estradiol concentrations were significantly reduced in the HH group at 18 weeks, in agreement with data obtained in one obese rat model [48]. The anti apoptotic role of estradiol [50] could possibly explain the significant increase observed in the number of atretic follicles. A significant increase in plasma leptin concentrations was only observed at 22 weeks of age. Unfortunately, these females were not put down nor their body composition analysed with TOBEC at that time, so it can only be assumed that this increase in leptin is related to higher percentage of fat in these animals. It is also difficult to try and relate this increased plasma leptin to direct effects on the ovary and more work is needed to elucidate this question. During gestation both progesterone and estradiol peaks tended to be delayed in HH does, and HH fetuses were growth retarded. In women, a positive correlation has been established between plasma progesterone concentrations and weight gain during pregnancy. In obese pubertal use, intra-uterine growth retardation has also been associated with a reduced placental secretion of progesterone [51]. No association was found, however, between gestational weight gain, maternal dietary fatty acid intake and estradiol concentrations [52].

Histological analysis of the ovaries of HH does at 18 weeks of age showed a higher number of atretic follicles and remnants of atretic follicles, indicating a possible increase in apoptotic mechanisms during folliculogenesis. Unfortunately, direct numbering of atretic follicle using specific - Terminal Transferase dUTP Nick End Labeling (TUNEL) on sections of these ovaries, did not display significative results (data not shown) as this assay is focused on on-going atresia and do not reveal ended previous processes evidenced in HES staining by the presence of fibrotic foci with central hypereosinophilic remnants of the zona pellucida. Moreover no difference was found in the expression of genes involved in the ovarian development. In parallel, histological analysis also showed a significantly reduced number of antral follicles in the HH group. Previous studies showed that the administration of a polyunsaturated fatty acids (PUFA) diet to cows during the periconceptional period reduces the number of small and middle size ovarian follicles, without affecting oocyte quality or *in vitro* cleavage [53]. In contrast, in a sheep model, short term overnutrition increased the number of large size ovarian follicles [54]. In agreement with the present study, rat models of obesity have impaired ovarian follicular growth with increased apoptosis [21], [55], but no difference in morphology nor in the number of antral follicles [21]. In the rabbit, the ovarian cortex is thin and is characterized by a small number of primordial and small developing follicles with a higher number of mature follicles [56], [57]. In terms of reproduction, rabbit females are characterized by the fact that mating induces ovulation. Follicular growth is a continuous process with waves of maturation that guarantee mature oocytes nearly anytime. During post-natal growth, secondary, tertiary and antral follicles appear between 4 and 12 weeks of age. Basal growth until antral formation is independent of gonadotrophin secretion whereas terminal follicular development depends on FSH. Therefore, the beginning of the study (10 weeks of age) occurred during a key period when hormonal dependence was starting, between tertiary and antral follicles and it is not known whether this was an important determinant in the observed results.

Systemic alterations associated with woman obesity (hyperinsulinemia, dyslipidemia, and symptoms of chronic inflammation) extend directly into the ovarian follicular microenvironment. Overweight and obese women were shown to exhibit elevated intrafollicular insulin, triglyceride and androgens which were associated with poor reproductive outcome [58], although there was no direct relationship between serum and follicular concentrations of free fatty acids [59]. Recently, a higher concentration of inflammatory factors was also observed in the follicular fluid of infertile obese women [60]. In the present study, the increased plasma cholesterol concentrations may have induced increased oxidative stress in the ovary, leading to increased follicular atresia. Whether direct effects of the maternal diet on the oocyte and/or effects on the oviductal and uterine environment induced fetal IUGR also remains to be determined, although the very early deregulation of gene expression in the embryo with the present model suggests that the oocyte quality may be affected by the HH diet [27].

In conclusion, this paper highlights, using an animal model, the possible adverse effects of unbalanced diets on the reproductive function and possible fertility of women. Although the diet used here is rich in poly-unsaturated fatty acids whereas the diet in humans consist mainly of saturated fats, this model remains relevant for hypercholesterolemia and also gives insight in general effects of high lipid diets. Given the dramatically increasing prevalence of obesity among women of reproductive age, it is essential that women be counseled on the reproductive risks of obesity and dangerous dietary behaviors and the proven benefits of lifestyle modification. This intervention must occur in the pre-conception period.

## Supporting Information

Data S1 **Sequences of qPCR primers.**
(DOC)

Data S2 **Weight according to age.** Mean ±SEM weight (kg) at 10, 13, 17, 23 and 27 weeks in the 2 groups (8 rabbits per groups).
(TIFF)

Data S3 **Serum cholesterol concentrations according to age.** Mean ±SEM serum cholesterol concentration (mmol/L) in the 2 groups according to age (13, 18, 22 and 27 weeks). ***P<0.001.
(TIFF)

Data S4 **Serum triglycerides concentrations according to age.** Mean ±SEM serum triglyceride concentration (mmol/L) in the 2 groups according to age (13, 18, 22 and 27 weeks).
(TIFF)

Data S5 **Body composition, organ weight and fat mass in rabbits at 18 weeks of age according to group.**
(DOC)

Data S6 **Gene expression in the 2 groups.** Relative expression of genes involved in folliculogenesis in the 2 groups.
(TIFF)

Data S7 **Biometric measurements made by ultrasound examination in fetuses from the 2 groups at 27 days of gestation.**
(DOC)
