# Supplementary material for: Dietary Lipid and Cholesterol Induce Ovarian Dysfunction and Abnormal LH Response to Stimulation in Rabbits
Source: PLoS One. 2013 May 14;8(5):e63101. doi: 10.1371/journal.pone.0063101 (PMC3653923; doi:10.1371/journal.pone.0063101)
Supplement: Data S1 — Sequences of qPCR primers. (DOC) [file pone.0063101.s001.doc]

| **Gene** | **Primers** |
| --- | --- |
| VASA | 5'-CAACTCGAGAGCTGATCAACCA-3' |
|  | 5'-CCCAACTGGGTTCCTCCATA-3' |
| HSD3B2 | 5'-GACGTCACAGGTGTCATTCAC-3' |
|  | 5'-GATCAGCACACTGGCTTGG-3' |
| FST | 5'-TCCTCAAGGCCAGGTGCAA-3' |
|  | 5'-GTGGAGCTGCCTGGACAGAA-3' |
| GDF9 | 5'-CTACAACACAGTTCGACTCTTCAC-3' |
|  | 5'-GTAACGCGATCCAGGTTAAACAG-3' |
| BMP15 | 5'-GCTGGTAAGGCCCTTGGCTAA-3' |
|  | 5'-TTGGTACGCTACCCGGTTTG-3' |
| ESR1 | 5'-GCACCCAGGGAAGCTTCTATT-3' |
|  | 5'-AGCCAGCAACATGTCAAAGATTT-3' |
| ESR2 | 5'-CTCACCAAGCTGGCTGACAA-3' |
|  | 5'-AGAGGCGCACTTGGTCCAA-3' |
| FOXL2 | 5'-TTTCCCCTTTCCCCCATCTG-3' |
|  | 5'-CTGAACCTTGCACCCAGCAT-3' |
| Caspase | 5'-CGGTTGAAGCCGACTTC-3' |
|  | 5'-GCACACAGGGACTGGAT-3' |
