# Supplementary figures and images for: Dietary Lipid and Cholesterol Induce Ovarian Dysfunction and Abnormal LH Response to Stimulation in Rabbits
Source: PLoS One. 2013 May 14;8(5):e63101. doi: 10.1371/journal.pone.0063101 (PMC3653923; doi:10.1371/journal.pone.0063101)

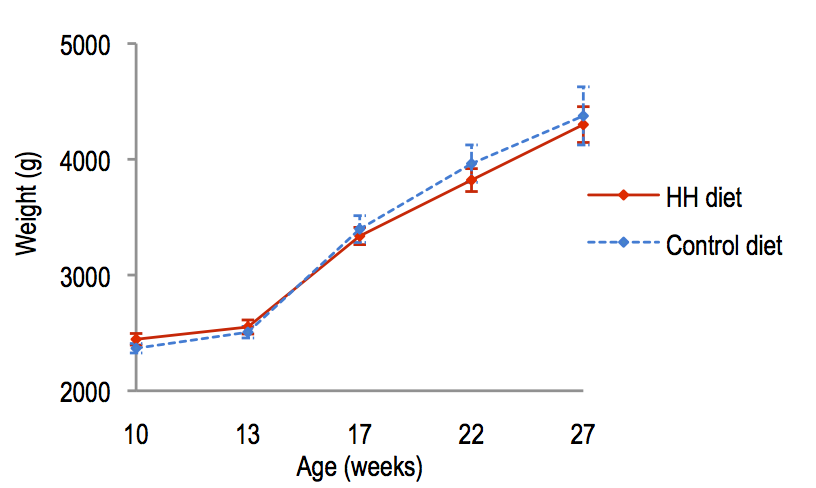

Supplement: Data S2 — Weight according to age. Mean ±SEM weight (kg) at 10, 13, 17, 23 and 27 weeks in the 2 groups (8 rabbits per groups). (TIFF) [file pone.0063101.s002.tif]

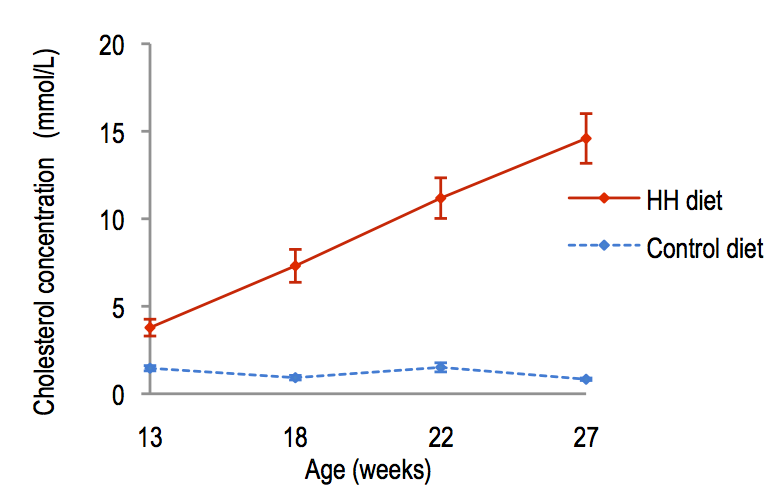

Supplement: Data S3 — Serum cholesterol concentrations according to age. Mean ±SEM serum cholesterol concentration (mmol/L) in the 2 groups according to age (13, 18, 22 and 27 weeks). ***P<0.001. (TIFF) [file pone.0063101.s003.tif]

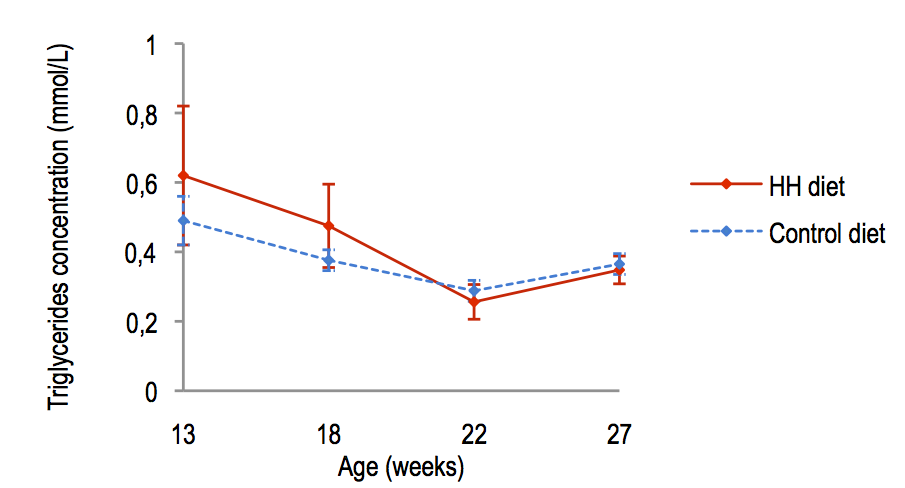

Supplement: Data S4 — Serum triglycerides concentrations according to age. Mean ±SEM serum triglyceride concentration (mmol/L) in the 2 groups according to age (13, 18, 22 and 27 weeks). (TIFF) [file pone.0063101.s004.tif]

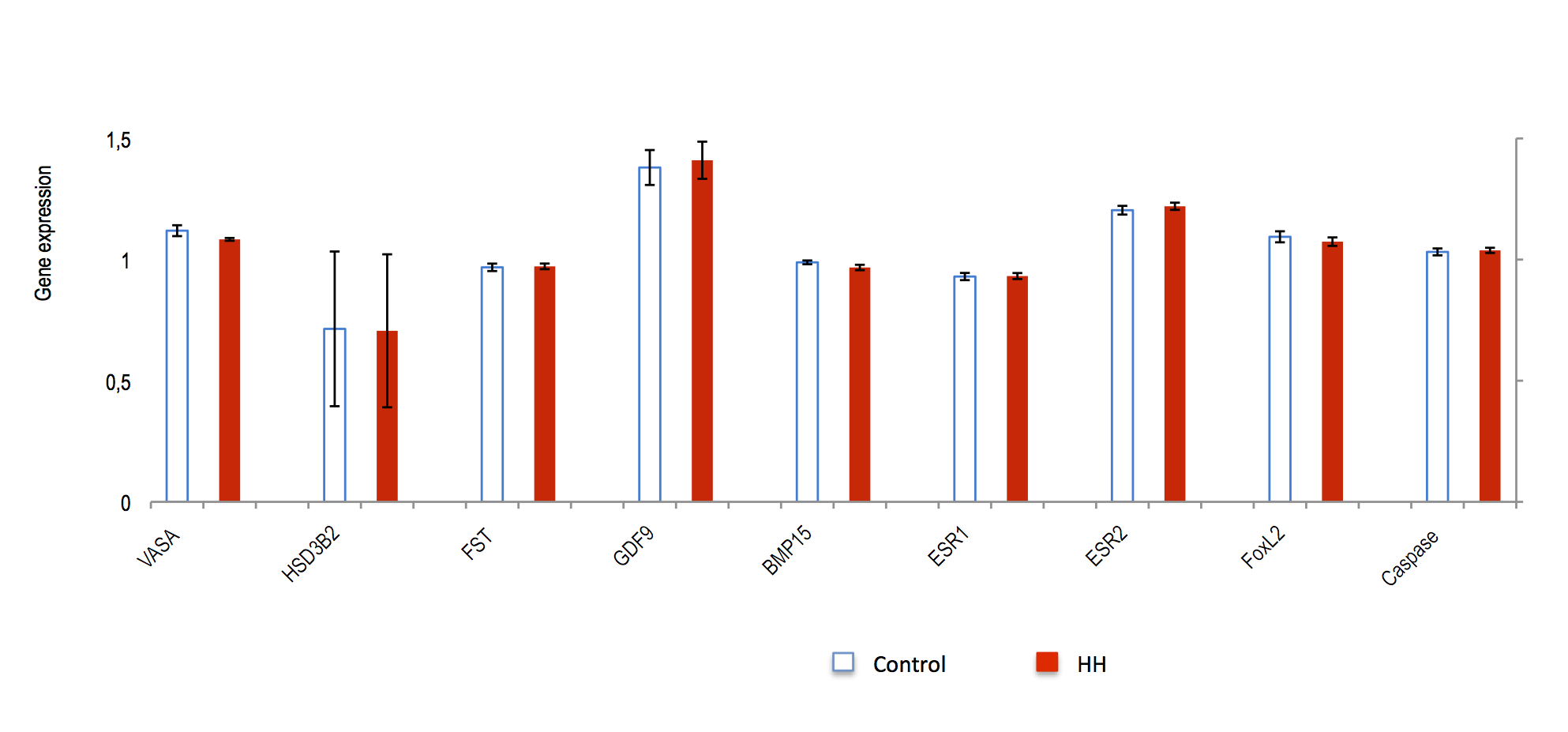

Supplement: Data S6 — Gene expression in the 2 groups. Relative expression of genes involved in folliculogenesis in the 2 groups. (TIFF) [file pone.0063101.s006.tif]
