# Supplementary material for: Dietary Lipid and Cholesterol Induce Ovarian Dysfunction and Abnormal LH Response to Stimulation in Rabbits
Source: PLoS One. 2013 May 14;8(5):e63101. doi: 10.1371/journal.pone.0063101 (PMC3653923; doi:10.1371/journal.pone.0063101)
Supplement: Data S5 — Body composition, organ weight and fat mass in rabbits at 18 weeks of age according to group. (DOC) [file pone.0063101.s005.doc]

|  |  | **HH diet** | **Control diet** | ***P*** |
| --- | --- | --- | --- | --- |
| **TOBEC results** | Water (%) | 68.11±0.58 | 69.51±0.44 | 0.04 |
|  | Lipid (%) | 6.11±0.19 | 5.67±0.14 | 0.04 |
|  | Energy (kJ/100g) | 746.23±17.76 | 706.73±13.62 | 0.05 |
| **Weight of organs** | Liver (g) | 108.74±8.28 | 111.69±7.23 | 0.40 |
|  | Kidney (g) | 16.83±1.58 | 16.00±0.80 | 0.32 |
|  | Ovaries (g) | 0.26±0.02 | 0.32±0.03 | 0.09 |
| **Weight of fat mass** | Interscapular fat (g) | 22.42±3.10 | 18.06±2.64 | 0.15 |
|  | Perirenal fat (g) | 79.42±13.63 | 64.40±14.25 | 0.23 |
|  | Total fat (g) | 101.84±15.41 | 82.95±16.90 | 0.20 |

Results of TOBEC are presented as mean ±SEM percentage of body composition.

Organ and fat mass weight are expressed in g.

Total fat is an approximation for the addition of interscapular and perirenal fat.
