# Supplementary material for: Dietary Lipid and Cholesterol Induce Ovarian Dysfunction and Abnormal LH Response to Stimulation in Rabbits
Source: PLoS One. 2013 May 14;8(5):e63101. doi: 10.1371/journal.pone.0063101 (PMC3653923; doi:10.1371/journal.pone.0063101)
Supplement: Data S7 — Biometric measurements made by ultrasound examination in fetuses from the 2 groups at 27 days of gestation. (DOC) [file pone.0063101.s007.doc]

| **Body part** | **Measure (unit)** | **HH diet** | **Control diet** | ***P*** |
| --- | --- | --- | --- | --- |
| Head | Length (mm) | 23.6 | 25.1 | 0.04 |
|  | Biparietal diameter (mm) | 12.6 | 13.2 | 0.012 |
|  | Surface (mm²) | 298.2 | 332.4 | 0.01 |
| Body | Length (mm) | 4.5 | 4.6 | 0.33 |
|  | Width (mm) | 1.8 | 2.0 | <0.01 |
|  | Surface (mm²) | 8.0 | 9.3 | <0.01 |
| Abdomen | Circumference (mm) | 69.7 | 78.9 | <0.01 |
| Femur | Length (mm) | 8.4 | 8.7 | 0.31 |

**7- Anthropometric measurement made by ultrasound examination in fetuses from the 2 groups at 27 days of gestation**
